# Natural allelic diversity of the calcium signaling regulators in plants

**DOI:** 10.1016/j.mocell.2024.100104

**Published:** 2024-08-02

**Authors:** Yejin Kim, Christian Danve M. Castroverde, Jong Hum Kim

**Affiliations:** 1 Department of Life Sciences, Pohang University of Science and Technology, Pohang 37673, Korea; 2 Department of Biology, Wilfrid Laurier University, Waterloo, Ontario N2L 3C5, Canada; 3 Institute for Convergence Research and Education in Advanced Technology, Yonsei University, Seoul 03722, Korea

**Keywords:** Calcium, Natural variation, Plant

## Abstract

Calcium ions act as secondary messengers in diverse signaling pathways in plants throughout their life cycle. Studies have revealed that calcium is involved in developmental events and in responses to external stimuli, such as biotic and abiotic stresses. Cellular calcium ion levels are tightly controlled by intricate molecular machinery such as calcium channels and pumps. Transient and spatial fluctuations in calcium levels are subsequently recognized by diverse calcium-decoding molecules, resulting in signal transduction. In this review, we highlight recent findings on natural variations in genes controlling calcium signaling in diverse plant biological processes. We then show how the calcium ion context is utilized by fine-tuning the natural variation in centrally important genes.

## INTRODUCTION

Natural allelic diversity of genes results from evolutionary processes, including artificial and natural selection, which confer phenotypic diversity (Alonso-Blanco et al., 2009). Natural variations provide valuable insights into the interactions between genes and the environment, particularly for traits related to plant adaptation (Alonso-Blanco et al., 2009). In addition, natural intraspecific variation can be leveraged toward the identification of gene functions, which has traditionally been achieved using artificially induced mutants in the laboratory (Alonso-Blanco and Koornneef, 2000, Alonso-Blanco et al., 2009). Because it underpins the genetic basis of the phenotypic diversity of plant adaptation to different environments (Alonso-Blanco et al., 2009), naturally occurring variation among crop wild relatives is utilized to improve cultivated crop plants (Bohra et al., 2022, Brozynska et al., 2015). Overall, this underscores the importance of natural variation for fundamental understanding and technological advancements.

Important targets for natural allelic diversity are components of critically sensitive signaling pathways. In plants, calcium is an important ion that serves as a nutrient and signaling molecule (Gilroy and Trewavas, 2001, Trewavas and Malho, 1998, White and Broadley, 2003). As signaling molecules, specialized transporters generate rapid fluctuations in cellular calcium ions (Luan and Wang, 2021). Changes in calcium levels are recognized by specific decoders, mostly calcium ion-binding proteins, to relay and amplify the initial cues (Luan and Wang, 2021). Numerous studies have shown that calcium signaling is involved in many important processes throughout the plant life cycle, including development (eg, flowering and pollen tube growth) and responses to environmental changes (eg, biotic and abiotic challenges) (Dodd et al., 2010, Helper and Wayne, 1985, Luan and Wang, 2021).

In this review, we summarize recent exciting findings regarding the natural variations in calcium signaling in plants under changing environments. We also highlight the naturally occurring fine-tuning of plant calcium signaling as a central module in adaptation to the surrounding environment. In this article, we review how natural variations fine-tune the context of calcium ions as hubs by regulating calcium transporters and decoders in response to diverse cues.

## MAIN TEXT

### Natural Variation in the Calcium Signaling Landscape in Plant Development

**Fig. 1:**
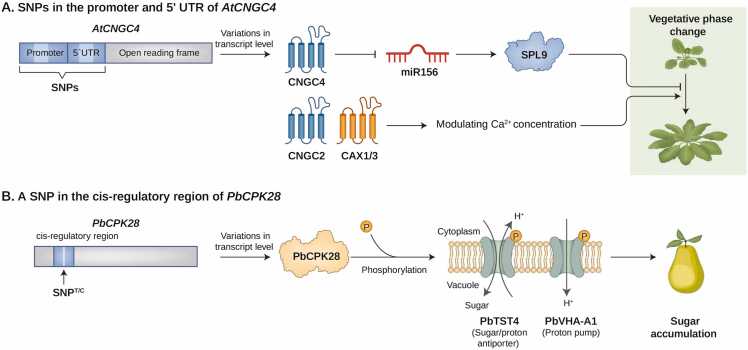
Natural variation of calcium signaling regulators in plant development. Variations in the transcriptional regulation of calcium channel-related genes can lead to differential transcript levels, contributing to developmental differences among *Arabidopsis* accessions. (A) SNPs in the 5′ UTR and promoter regions of *CNGC4* led to differences in *CNGC4* transcript abundance. The encoded CNGC4 calcium channel eventually leads to transcriptional repression of *miR156* and *SPL9*, the master regulators of VPC. Increased transcript levels of *miR156* and *SPL9* can delay the VPC phenotype. The vacuolar calcium transporters, CAX1/3, are also involved in modulating plant cell Ca^2+^ concentrations and promoting VPC. (B) SNP^T/C^ in the cis-regulatory region of *PbCPK28* result in differential expression of *PbCPK28*. The encoded kinase PbCPK28 phosphorylates PbTST4 and PbVHA-A1, thereby enhancing sugar transport into the vacuole. AtCNGC4, Arabidopsis thaliana cyclic nucleotide-gated ion channel 4; CAX1/3, cation exchanger 1/3; miR156, microRNA 156; SNP, single-nucleotide polymorphism; SPL9, squamosa promoter binding protein-like 9; UTR, untranslated region.

Recent findings have highlighted that natural variations in calcium channels and calcium-channel-related genes are important for plant development. For example, plants undergo a vegetative phase change (VPC), which is a developmental shift from the juvenile to adult phase of plant growth (Poethig and Fouracre, 2024). External calcium concentrations significantly influence VPC regulation, as evidenced by the delayed VPC phenotype observed under low calcium ion conditions (Feng et al., 2016). A recent study revealed that cellular calcium levels coordinated by calcium transporters are involved in the VPC process in *Arabidopsis* (Wang et al., 2024). A genome-wide association study (GWAS) identified 2 significant quantitative trait loci for VPC in *Arabidopsis* and the *Cyclic nucleotide*–*gated ion channel* (C*NGC4*) gene was further investigated in a recent study (Wang et al., 2024). Disruption of *CNGC4* expression, as observed in the *cngc4* knockout mutant, results in delayed VPC and elevated levels of microRNA 156 (*miR156)* and *Squamosa promoter binding protein-like 9* transcripts, which are master regulators of VPC, resulting in a delayed VPC phenotype (Wang et al., 2024, Wu et al., 2009). Further studies revealed that *CNGC4* and other calcium channel genes, such as *CNGC2*, which encodes a plasma membrane calcium transporter essential for floral development and pathogen-induced defense mechanisms, and *Cation exchanger 1/3*, which encodes vacuolar calcium transporters, promote VPC in *Arabidopsis* (Chan et al., 2003, Chin et al., 2013, Punshon et al., 2012, Tian et al., 2019, Wang et al., 2024). Notably, the authors identified that natural variations in the promoter and 5′ untranslated region (UTR) region of *CNGC4* result in differential gene expression in *Arabidopsis* accessions, thereby causing variation in VPCs (Fig. 1A; Wang et al., 2024). For example, the promoter activity of *CNGC4*^Col-0^ (haplotype A, found in accessions such as Col, Ost-0, and IP-Ber-0) was lower than that of *CNGC4*^Tu-B1-2^ (haplotype B, found in accessions such as Tu-B1-2, TDr-9, and Ang-0) (Wang et al., 2024). Because the geographical distributions of the 2 haplotypes were different (haplotype A at higher latitudes and haplotype B at lower latitudes), the *CNGC4* alleles may have been preserved by the selection pressure of the local environment (Wang et al., 2024).

Another prominent example is the natural variation in calcium-dependent protein kinases that contribute to sugar content diversity in pear accessions (Li et al., 2023). Sugars, which are essential for plant growth, development, metabolite synthesis, and stress tolerance, are stored in vacuoles. Sugar content is greatly influenced by environmental factors and exhibits different plasticity among plant species (Wormit et al., 2006). In pears (*Pyrus bretschneideri*), a calcium-dependent protein kinase (PbCPK28) plays a crucial role in sugar accumulation by interacting with the vacuolar sugar transporter protein Tonoplast membrane Sugar Transporter 4 (PbTST4) and the vacuolar proton pump Vacuolar-type H^+^-ATPases-A1 (PbVHA-A1) (Li et al., 2023). Specifically, PbCPK28 phosphorylates PbTST4, which functions as a sugar-proton antiporter that simultaneously facilitates sugar import and proton export from the vacuoles (Fig. 1B; Li et al., 2023). The phosphorylated PbTST4 residue is important for transport capacity, and its transport function is decreased in PbTST4 phosphorylation-site mutants (Li et al., 2023). In addition, PbCPK28 phosphorylates PbVHA-A1*,* leading to increased ATPase activity of PbVHA-A1 and providing a pH gradient between the vacuole and cytosol (Li et al., 2023). Indeed, the overexpression of PbVHA-A1 enhanced fructose accumulation when transiently expressed in pear fruits (Li et al., 2023). Interestingly, *PbCPK28* has a SNP^T/C^ (SNP13^C^: lower expression of *PbCPK28*, resulting in lower fructose accumulation; SNP13^T^: higher expression of *PbCPK28*, resulting in higher fructose accumulation) in its cis-regulatory region, leading to differential expression levels of *PbCPK28* among pear accessions, thereby contributing to the intraspecies diversity of sugar content in pears (Li et al., 2023). Taken together, natural allelic variations in calcium encoder and decoder genes contribute to the profound differences in developmental phenotypes observed in important models and crop plant species.

### Natural Variation in the Calcium Signaling Landscape in Plant Responses to Biotic Challenges

As key signaling molecules, calcium ions play a critical role in plant immunity. Many studies have shown the role of calcium ions and calcium regulatory modules in plant immune signaling and responses (see the recent review papers: Koster et al., 2022; Wang and Luan, 2024). In this section, we discuss the reported natural variations in genes regulating the calcium ion context of plant immunity.

*Accelerated cell death 6* (*ACD6*), which encodes a transmembrane protein with intracellular ankyrin domains, was originally cloned from the dominant gain-of-function mutant *acd6-1* (Lu et al., 2003). Using forward genetic screening to identify genes involved in plant pathogen defense, the *acd6-1* mutant was found to carry a single amino acid substitution in the transmembrane domain (Lu et al., 2003, Rate et al., 1999). Further analysis revealed that *acd6-1* exhibits key features of autoimmunity, such as smaller biomass, autonomous cell death, activated defense-related gene expression, enhanced accumulation of the phytohormone salicylic acid (SA) and the antimicrobial metabolite camalexin, and resistance against pathogens, indicating that ACD6 is a crucial regulator of plant defense against pathogens (Lu et al., 2003, Rate et al., 1999).

Two recent studies elucidated the biochemical functions of ACD6-like proteins as calcium channels in plants. In the first report, the wheat *Leaf rust resistant gene 14a* (*Lr14a*), which was identified in the Swiss winter wheat cultivar Forno and confers resistance to the leaf rust fungal pathogen *Puccinia triticina*, was cloned (Kolodziej et al., 2021). The cloned gene encodes a protein of the Ankyrin Repeat-TransMembrane gene family, harboring putative ankyrin and transmembrane domains, such as ACD6 in *Arabidopsis* (Kolodziej et al., 2021). A striking feature of Lr14a is its considerable structural similarity to the animal transient receptor potential ankyrin 1 ion channel, a heat/pain-induced calcium channel, despite its low amino acid sequence similarity (Kolodziej et al., 2021). Green fluorescent protein-tagged Lr14a localizes to the plasma membrane, and ectopic expression of *Lr14a* results in a water-soaking-like phenotype in *Nicotiana benthamiana* leaves, which is suppressed by calcium channel blocker LaCl_3_ treatment (Kolodziej et al., 2021). This study provides the first clue that Lr14a may act as a calcium channel. In another study, the ectopic expression of *Arabidopsis* ACD6 in *Xenopus oocytes* and human embryonic kidney cells led to enhanced calcium ion influx in both cell types, indicating that ACD6 is a calcium ion-conducting channel (Chen et al., 2023). These results link the role of calcium ions to the altered growth and immunity observed in *ACD6* loss- and gain-of-function mutants (Lu et al., 2003, Rate et al., 1999).

**Fig. 2:**
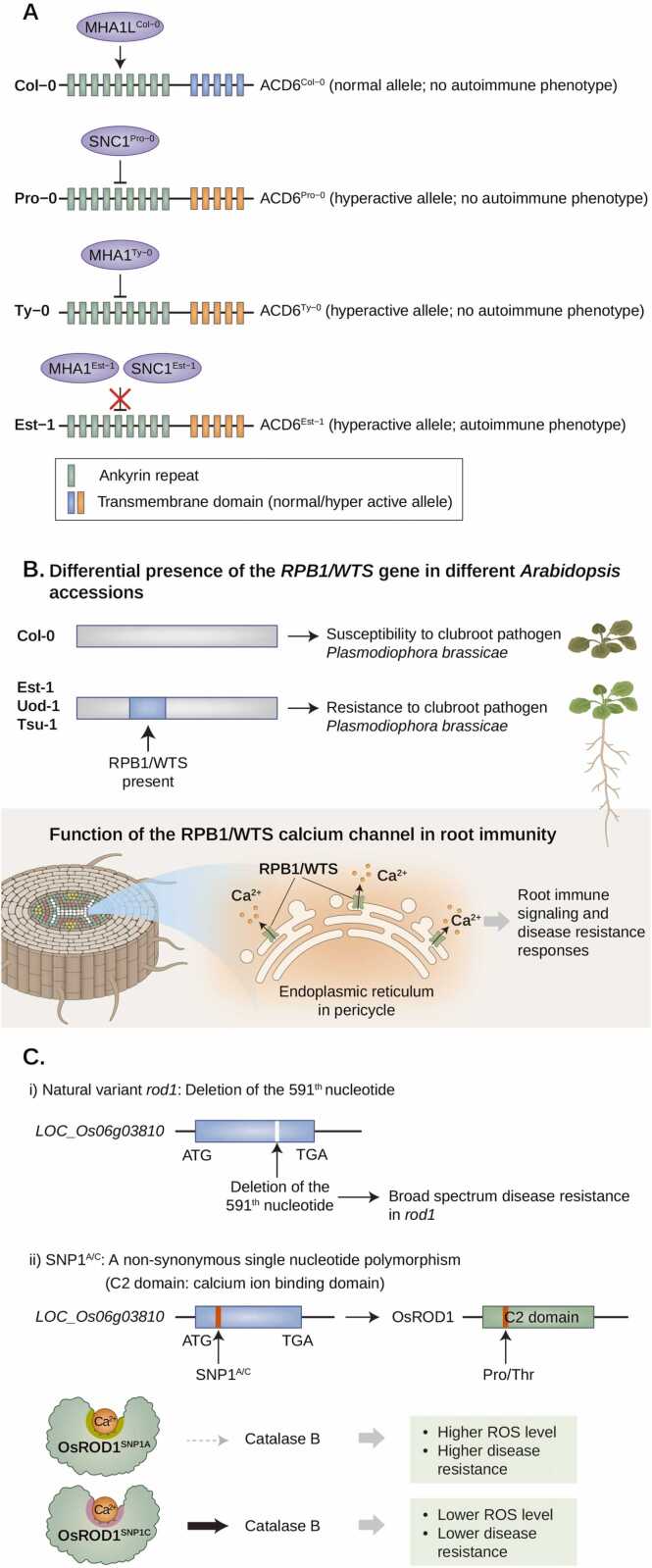
Natural variation in calcium-signaling regulators in plant adaptation to biotic stress challenges. (A) Illustration describing the natural variation in ACD6 and its regulators SNC1 and MHA1L in *Arabidopsis* Col-0, Pro-0, Est-1, and Ty-0 ecotypes. In Col-0, MHA1L^Col-0^ potentiates the activity of ACD6^Col-0^ (normal allele) in response to pathogen challenge. Conversely, Pro-0 and Ty-0 accessions possess the hyperactive ACD6^Pro-0^ or ACD6^Ty-0^ allele, which is suppressed by SNC1^Pro-0^ or MHAL1^Ty-0^ resulting in a nonautoimmune phenotype. Finally, Est-1 shows an autoimmune phenotype because SNC1^Est-1^ and MHA1L^Est-1^ are unable to suppress hyperactive ACD6^Est-1^. (B) Natural variation in the larger RPB1 genomic region in *Arabidopsis*. Clubroot pathogen-susceptible accessions (eg, Col-0) do not contain the RPB1/WTS gene, whereas clubroot pathogen-resistant accessions (eg, Est-1, Uod-1, Tsu-1) possess RPB1/WTS. The encoded RPB1/WTS protein is a putative calcium channel expressed in the ER of pericycle tissues, effectively inducing root immune responses against *P brassicae* to safeguard vascular tissues from pathogen invasion. (C) Natural variation in *OsROD1* gene locus in rice*.* (i) Deletion of the nucleotide 591 of *OsROD1* results in loss-of-function of *OsROD1*, which confers broad-spectrum disease resistance in rice varieties harboring the *rod1* variant. (ii) A nonsynonymous single nucleotide polymorphism SNP1^A/C^ distinguishes disease-resistance and susceptible rice varieties. SNP1^A/C^ of *OsROD1* generates a Pro-to-Thr substitution in the C2 domain of OsROD1. OsROD1^SNP1A^ activates OsCatB less effectively than OsROD1^SNP1C^, resulting in higher ROS levels and disease resistance under pathogen challenge. ATG, start codon; ER, endoplasmic reticulum; OsCatB, Oryza sativa Catalase B; OsROD1, Oryza sativa resistance of rice to diseases 1; TGA, stop codon.

Recent findings have helped expand our conceptual understanding of the natural variation in the *ACD6* locus as a factor that balances the trade-off between growth and defense via calcium homeostasis in *Arabidopsis*. One study identified the *ACD6* locus as the causative gene of the autoimmune phenotype in the Est-1 ecotype, an *Arabidopsis* accession with slow leaf production and necrosis, using a quantitative trait mapping approach (Fig. 2A; Todesco et al., 2010). Similar to the *acd6-1* gain-of-function mutant in Col-0, the Est-1 ecotype showed a skewed growth/defense balance toward enhanced defense (Fig. 2A; Todesco et al., 2010). Interestingly, the *ACD6*^Est-1^ allele is also a hyperactive allele sufficient to induce an autoimmune phenotype in a Col-0 background (Todesco et al., 2010). Further analysis revealed additional hyperactive alleles of *ACD6* gene in wild accessions of *Arabidopsis* (found in ∼10% of *Arabidopsis thaliana* wild strains), indicating that allelic diversity at *ACD6* gene locus is utilized among natural *Arabidopsis* strains to balance the trade-off between growth and defense (Todesco et al., 2010). The allelic diversity in the *ACD6* gene may be a product of calcium homeostasis through fine-tuning of ACD6 calcium channel activity in response to the surrounding environment of wild *Arabidopsis* strains. In addition, natural variations in *Lr14a* have been established for immunity in various wheat cultivars, indicating that fine-tuning calcium homeostasis is a natural strategy for controlling plant immunity (Kolodziej et al., 2021).

In addition, previous reports have identified natural variations in the *ACD6* gene and in its second-site modifiers to fine-tune ACD6-mediated calcium homeostasis (Fig. 2A; Chen et al., 2023, Zhu et al., 2018). The first example is *Suppressor of NPR1-1, constitutive 1* (*SNC1*), a modifier of ACD6 activity in *Arabidopsis* (Zhu et al., 2018). Some *Arabidopsis* accessions harboring hyperactive ACD6 alleles do not show autoimmunity, implying the presence of extragenic suppressors in these accessions (Zhu et al., 2018). Indeed, a GWAS revealed that the gene cluster *Recognition of Peronospora parasitica 4/5*, which comprises important alleles that mediate resistance to the oomycete pathogen *Hyaloperonospora arabidopsidis*, is one of the extragenic suppressors of *ACD6*^Est-1^ (Zhu et al., 2018). Within this gene cluster, natural variations in *SNC1* contribute to the autoimmunity of the hyperactive ACD6^Est-1^ allele (Zhu et al., 2018). For example, the introduction of the hyperactive *SNC1*^Est-1^ allele into Pro-0, which also harbors a hyperactive ACD6 allele (but no autoimmunity), resulted in a necrotic phenotype (Zhu et al., 2018).

In addition, another GWAS approach using *Arabidopsis* ecotypes harboring the hyperactive ACD6 allele revealed another type of modulator called *Modifier of Hyperactive ACD6 1* (*MHA1*) and its paralog *Modifier of Hyperactive ACD6 1*-Like (*MHA1L*), which encodes peptide ligands that regulate ACD6 activity (Fig. 2A; Chen et al., 2023). Notably, although MHA1 is a second-site suppressor of hyperactive ACD6^Est-1^, MHA1L works as an activator of the normal *ACD6*^Col-0^ allele, indicating that the gene pairs comprising hyperactive *ACD6* allele*-MHA1* and normal *ACD6* allele*-MHA1L* could be utilized based on the context of each ecotype (Chen et al., 2023). This study is the first to demonstrate ion channel activity for a plant ankyrin domain protein as well as its regulation by peptide ligands, similar to the well-studied TRP ion channel regulation in animals (Lin King et al., 2019). This finding opens new avenues for research on plant immunity and could be of interest to those studying ligand-controlled ankyrin proteins in animals.

Another example of a potential calcium channel identified through GWAS is resistance to *Plasmodiophora brassicae* 1 (*RPB1*) or *WeiTsing* (*WTS*) (Fig. 2B; Ochoa et al., 2023; Wang et al., 2023), which is crucial in conferring disease resistance to the devastating clubroot pathogen, *P brassicae*. Using a panel of 142 *Arabidopsis* accessions, effective clubroot resistance was identified in 11 accessions, including the autoimmune ecotype Est-1, which was instrumental in *ACD6* discovery (Ochoa et al., 2023). Of the 2 loci of interest, *RPB1*/*WTS* emerged as the causative gene, as evidenced by both knockout analyses of clubroot-resistant accessions and transgenic complementation of the clubroot-susceptible accession Col-0 (Ochoa et al., 2023). *RPB1/WTS* expression is induced by *P brassicae* infection and the encoded protein is localized in the endoplasmic reticulum (Wang et al., 2023). Further mechanistic analyses showed that RPB1/WTS is a calcium-permeable cation-selective channel, which is an important immune signaling component downstream of pathogen recognition (Wang et al., 2023). Unlike ACD6 and most other immunity-related calcium channels that lead to cytoplasmic calcium influx from the extracellular environment, RPB1/WTS releases intracellular calcium stores from the endoplasmic reticulum into the cytoplasm during immune activation (Wang et al., 2023). Due to its tissue-specific expression in the pericycle (the outermost boundary of the stele), RPB1/WTS activation effectively induces immune responses by protecting vascular tissues from clubroot infections (Wang et al., 2023). Because numerous soil-borne pathogens cause plant pathogenesis by breaching the stele, it would be interesting to investigate whether the RPB1/WTS function provides broad-spectrum resistance against most, if not all, root-invading pathogens.

Apart from calcium encoders such as ACD6 and its modulating peptide ligands, a recent study showed an example of natural variation in calcium decoder proteins in plant immunity. In rice, a recently identified recessive disease resistance gene variant called *resistance of rice to diseases1* (*rod1*) confers broad-spectrum resistance to rice blast, sheath blight, and bacterial blight, but also results in a growth penalty (Fig. 2C; Gao et al., 2021). Subsequent map-based cloning revealed that *ROD1* encodes a protein containing a C2 domain with a recessive *rod1* variant containing a single-nucleotide deletion that causes a frameshift mutation (Gao et al., 2021). Essential for the function of ROD1 in immunity, the C2 domain directly binds Ca^2+^ ions to enable ROD1 association with phospholipids and plasma membrane localization (Gao et al., 2021). Further characterization has shown that ROD1 interacts with Catalase B (CatB), which enhances ROD1 localization to the plasma membrane and CatB-mediated reactive oxygen species (ROS)-scavenging activity (Gao et al., 2021). Notably, a GWAS of 262 Asian cultivated rice accessions identified a single-nucleotide polymorphism (SNP) within the ROD1 coding region, generating a proline-to-threonine amino acid substitution that is notably associated with disease resistance (Gao et al., 2021). ROD1 harboring SNP1^A^ is less effective in promoting the scavenging activity of CatB, resulting in a relatively stronger resistance than SNP1^C^ (Gao et al., 2021)_._ SNP1^A^ is predominantly retained in indica strains, whereas SNP1^C^ is mainly found in wild rice and japonica strains, implying that natural variations in the calcium signal decoder ROD1 fine-tune ROS homeostasis to confer rice strain-specific disease resistance (Gao et al., 2021). This example shows how artificial and natural selection pressures diversify natural variants of the calcium decoder ROD1 (*rod1*: loss-of-function allele; SNP within the ROD1 coding region: fine-tuning of ROD1 activity) to control the calcium-mediated growth/defense tradeoff in rice.

Another example revealed the natural variation in calcium decoders in response to insect challenges in soybeans (Wang et al., 2022). A gene encoding *Glycine max* calcium-dependent protein kinase 17 was isolated from quantitative trait locus analysis to identify genes involved in defense against insects in soybean (Wang et al., 2022). Further studies showed that knockdown plants or plants overexpressing *G max* calcium-dependent protein kinase 17 showed enhanced susceptibility or resistance to common cutworms, respectively. Sequence analysis of the soybean accessions identified 6 and 7 haplotypes from the cultivated and wild soybean accessions, respectively. The haplotypes 1, 2, and 3 (Hap1, 2, and 3) were identified from wild accessions, and larval weights fed on Hap2 and 3 were heavier than those fed on Hap1, indicating that Hap 1 is a resistant haplotype (Wang et al., 2022).

Overall, these representative studies on the genetic variation in calcium-generating channels and calcium-sensing regulators underscore the functional significance of distinct alleles in shaping the calcium ion context of plant immune signaling and responses in various plant taxa.

### Natural Variation in the Calcium Signaling Landscape in Plant Responses to Abiotic Challenges

Calcium signaling is crucial for modulating abiotic stress responses in plants (Knight, 2000). Indeed, several studies have revealed that natural variations in calcium channels and calcium-related genes enable plants to respond differently to various abiotic stressors.

Natural variation in the promoter region of the calcium decoder gene *Oryza sativa calcineurin B-like protein 10* (*OsCBL10*) is involved in the differential response to flooding stress by modulating calcium influx in rice (Ye et al., 2018). Ca^2+^ functions as a secondary messenger during hypoxic signaling, which can activate downstream cascades to promote germination under flooding stress (Ye et al., 2018). Two different ecotypes of rice, flood-intolerant Up221 and flood-tolerant Low88, exhibit variations in their resistance to flooding stress (Ye et al., 2018). Under flooding stress conditions, Up221 exhibited a higher Ca^2+^ influx rate in the coleoptile tips and showed greater fluctuations in net calcium levels than Low88, suggesting that calcium ions are involved in germination during flooding in rice (Ye et al., 2018). Further experiments revealed that cyclosporin A (a calcineurin B-like (CBL) inhibitor), but not trifluoperazine (a calmodulin inhibitor), recovered the suppressed shoot growth under flooding stress conditions in Up221, indicating that CBLs are involved in susceptibility to flooding-intolerant ecotypes (Ye et al., 2018). These cultivars demonstrated differential expression levels (higher in Low88) of the *OsCBL* genes, which encode calcium-binding proteins involved in cytosolic Ca^2+^ decoding (Ye et al., 2018). These 2 cultivars differ in the promoter sequences of *OsCBL10*, particularly in regions where regulatory promoters are located, potentially resulting in differential regulation of gene expression (Ye et al., 2018). Notably, natural variations in *OsCBL10* promoter sequences have been found in various rice cultivars beyond Up221 and Low88 (Ye et al., 2018). Flooding-tolerant cultivars possess the flooding-tolerant type (T-type) promoter of *OsCBL10* and have shown lower Ca^2+^ flow and higher α-amylase activities that are crucial in hydrolyzing native starch granules in the endosperm, enabling plants to better respond to flooding stress (Ye et al., 2018). In contrast, flood-intolerant cultivars have a flood-intolerant (I-type) promoter, and these cultivars exhibit higher calcium fluctuations than those with T-type promoters (Ye et al., 2018). The T-type promoter variant is exclusively found in lowland japonica cultivars, whereas the I-type promoter variant is found in upland japonica, upland indica, and lowland indica cultivars (Ye et al., 2018). Thus, natural variations in the *OsCBL10* promoter are associated with divergent calcium signaling-mediated responses to flooding stress in different rice ecotypes (Ye et al., 2018).

**Fig. 3:**
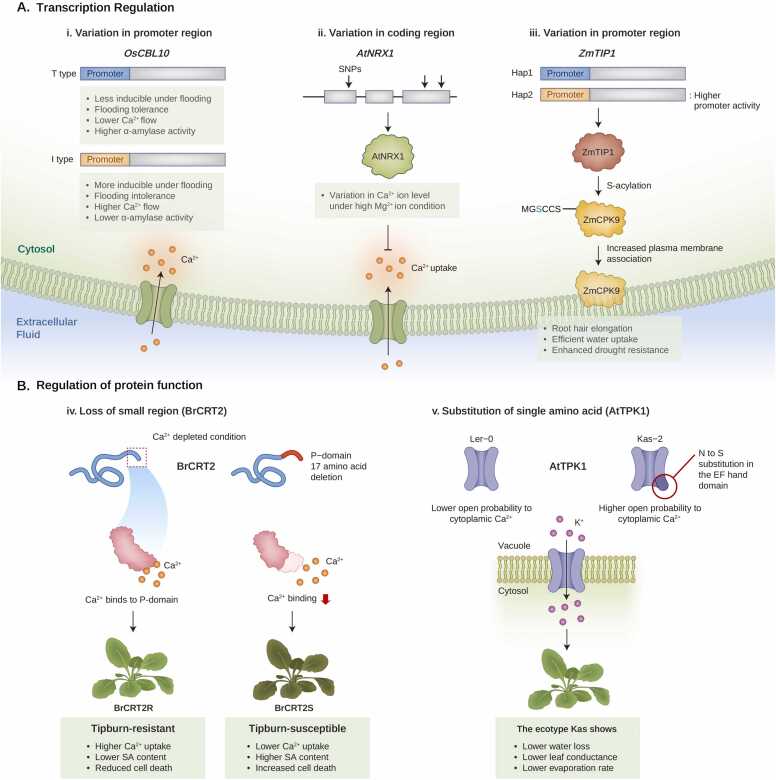
Natural variation in calcium-signaling regulators in plant adaptation to abiotic stress conditions. (A) Natural variation in the promoter region of calcium channel genes leads to differential responses to abiotic stress by regulating gene expression levels and protein function. (i) The *OsCBL10* gene in 2 ecotypes (Up221 and Low88) has different types of promoters, which have variations in the promoter regulatory region. This leads to differential *OsCBL10* expression levels, which is higher in Up221 (with the I-type promoter). Flooding-tolerant cultivars including Low88 show weak promoter activity of *OsCBL10*, lower calcium flow and higher α-amylase activity, whereas flooding-intolerant cultivars including Up221 show stronger promoter activity of *OsCBL10*, higher calcium flow, and lower α-amylase activity. (ii) The SNP variations in the *AtNRX1* coding region resulting variations of calcium influx under high Mg^+^ stress. AtNRX1 can negatively regulate Ca^2+^ uptake under Mg^2+^ stress. (iii) Two variations of the *ZmTIP1* gene (*Hap1* and *Hap2*) have differences in the promoter region. *Hap2* has a higher promoter activity, which leads to higher expression of *ZmTIP1*. The ZmTIP1 protein modifies ZmCPK9 by S-acylation, where the cysteine residue of the ZmCPK9 N-terminal MGSCCS motif is modified. This modification promotes ZmCPK9 association with the plasma membrane, leading to enhanced root hair growth and efficient water uptake under drought. (B) Natural variations in protein sequences lead to differential protein function and response to calcium ion stress conditions. (iv) Two natural variations (*BrCRT2R* and *BrCRT2S*) have different resistance to tipburn disease, and the expression level of *BrCRT2* is higher in *BrCRT2R*. In contrast, *BrCRT2S* has an allele with a 51-bp deletion in the eighth exon, leading to the loss of 17 amino acids in the BrCRT2 P domain. The Ca^2+^ binds to the P domain, which enables higher calcium ion uptake and storage. As BrCRT2S lines carry the P domain deletion, the Ca^2+^ supply becomes insufficient to cause tipburn disease. BrCRT2 also functions as a repressor of SA biosynthesis and reduces cell death. (v) The variation in the *AtTPK1* allele in 2 natural accessions (Ler-0 and Kas-2) shows differential calcium dependence. Along with the difference in biochemical characteristics, the ecotype Kas-2 shows lower water loss, leaf conductance, and evaporation rate contrast to Ler-0. BrCRT2, Brassica rapa Calreticulin 2; SNP, single-nucleotide polymorphism.

Natural variations in genes regulating Ca^2+^ uptake also cause different responses to stress from inorganic ions, such as Na^+^ and Mg^2+^. Natural variation in *Arabidopsis* nucleoredoxin (*AtNRX1*) expression among diverse *Arabidopsis* ecotypes is an example of adaptation to serpentine soils containing high levels of Mg^2+^ ions (Niu et al., 2018). Although plants require sufficient Mg^2+^ for optimal growth and development, excessive accumulation of Mg^2+^ typically results in cellular toxicity (Brady et al., 2005). Under high Mg^2+^ conditions, the resulting accumulation of ROS (eg, hydroxyl radicals) can damage proteins, RNA, and DNA (Brady et al., 2005) and cellular ROS accumulation can activate calcium channels in the plasma membrane and increase the tip-focused calcium concentration in the cytosol (Brady et al., 2005). The identification of SNPs related to Ca^2+^ uptake under high Mg^2+^ using GWAS revealed a strong peak of SNP variations in the *AtNRX1* gene, a calcium binding (CD)-domain-containing protein (Niu et al., 2018). AtNRX1 is a negative regulator of Ca^2+^ uptake under high Mg^2+^ conditions and influences cytosolic Ca^2+^ concentrations in plant cells (Fig. 3A). In parallel with a previous study, loss-of-function *atnrx1* mutants showed enhanced Ca^2+^ uptake under high Mg^2+^ conditions (Kneeshaw et al., 2017, Niu et al., 2018). SNPs in *AtNRX1* modulate Mg^2+^ availability by affecting the Ca^2+^ uptake capacity of AtNRX1. Consequently, variations in Mg^2+^ availability led to differences in root development, rosette growth, and hypocotyl length across the various ecotypes. NRX1 also removed ROS and regulated cytosolic Ca^2+^ concentrations (Fig. 3A). This indicates that the differential expression of *AtNRX1* through SNP variations in the *AtNRX1* gene is utilized by diverse ecotypes of *Arabidopsis* under dynamically fluctuating natural conditions (Niu et al., 2018).

Furthermore, the differential expression of a post-translational regulator of the canonical calcium signaling molecule functions in the drought response in maize (Wang et al., 2016, Zhang et al., 2020). In a previous study, an SNP in the promoter region of maize *Tip Growth Defective 1* (*ZmTIP1*) was significantly associated with drought tolerance in maize seedlings (Wang et al., 2016). Two distinct haplotypes of the *ZmTIP1* gene, Hap1, and Hap2, exhibit variations in their promoter regions (Zhang et al., 2020). In particular, the *Hap2* promoter variant led to higher promoter activity, resulting in increased root hair length and higher *ZmTIP1* expression levels in lines harboring *Hap2* (Zhang et al., 2020). Further investigation revealed that ZmTIP1 acts as an S-acyltransferase that targets ZmCPK9 for S-acylation of its cysteine residues within the N-terminal MGSCCS motif (Fig. 3A; Zhang et al., 2020). By mediating the S-acylation of ZmCPK9, ZmTIP1 can facilitate the association between ZmCPK9 and the plasma membrane, thereby enhancing polar cell growth, facilitating efficient water uptake, and enhancing drought tolerance (Zhang et al., 2020).

In addition to allelic diversity in gene promoter sequences, natural variation in coding regions leads to differential regulation of protein functions, differentiating the responses of various ecotypes to calcium-depleted conditions by modulating calcium uptake and storage. This is exemplified by a Ca^2+^-related disorder in Chinese cabbage (*Brassica rapa*) called tipburn, which is caused by an insufficient Ca^2+^ supply, resulting in the collapse and necrotic cell death of actively growing leaf apices and margins (Collier and Tibbitts, 1982). The Calreticulin gene Brassica rapa Calreticulin 2 (*BrCRT2)* plays a central role in mediating tipburn resistance in Chinese cabbage by regulating calcium uptake (Su et al., 2019). Chinese cabbage has 2 natural varieties, BrCRT2R (tipburn-resistant) and BrCRT2S (tipburn-susceptible), with higher expression of the *BrCRT2* gene observed in the BrCRT2R variety (Su et al., 2019). The tipburn-susceptible lines carried an allele with a 51-bp deletion in exon 8, leading to the truncation of 17 amino acids in the BrCRT2 P domain (Fig. 3B; Su et al., 2019). The P domain is important because it contains a high-affinity EF hand-like structure for calcium binding (Michalak et al., 2009). Under normal and calcium-deficient conditions, BrCRT2R exhibits a higher calcium uptake capacity and calcium storage than BrCRT2S (Su et al., 2019). Crosstalk with defense hormones is also crucial because SA signaling and cell death are related to tipburn disease (Su et al., 2019). Under calcium-depleted conditions, SA biosynthesis increases (Su et al., 2019). When comparing the SA content of the 2 BrCRT2 variants under calcium-depleted conditions, the SA content was lower in BrCRT2R, indicating that CRT2 represses SA biosynthesis and reduces cell death (Su et al., 2019).

An example of a natural allelic variation affecting protein function is *A thaliana* two-pore K^+^ 1 (Hartley and Maathuis, 2016). Two Pore K^+^ (TPK) is a potassium-selective ion channel involved in defense responses against abiotic stresses such as stomatal closure (Kwak et al., 2008). Calcium ions and cytoplasmic factors regulate the activity of the TPK proteins, which are K^+^-selective ion channels that mediate vacuolar K^+^ efflux into cells (Hartley and Maathuis, 2016). Through sequence analysis, natural allelic nonsynonymous variations were identified in the fourth transmembrane domain and first EF-hand, which are present in the cytoplasmic C-terminus of AtTPK1 Kas-2 ecotypes (Hartley and Maathuis, 2016). Although single-channel conductance and K^+^:Na^+^ selectivity did not differ between the Ler and Kas genotypes, the sensitivity of TPK1 opening probability to cytoplasmic Ca^2+^ is different between Ler and Kas (Hartley and Maathuis, 2016). One of the resulting SNPs is the C-terminal EF-hand motif, where the asparagine side group is replaced by a serine side group (N295S) in the Kas-2 genotype, implying that the amino acid change may affect the interaction between the EF-motif and the channel gate (Hartley and Maathuis, 2016). Physiological analysis further revealed that the leaves of Ler-0 exhibited a higher rate of water loss and greater leaf conductance than Kas-2 leaves, and the evaporation rate in Ler leaves was higher than that in Kas-2 leaves, likely because of the larger stomatal aperture in Ler-0 (Hartley and Maathuis, 2016).

Collectively, variations in the regulatory and coding regions of genes essential for calcium signaling pathways are significant drivers of diversity in plant tolerance and/or susceptibility to numerous abiotic stressors.

## CONCLUSIONS AND FUTURE PERSPECTIVES

Our review highlights recent studies on natural variations in the fine-tuning of calcium-mediated signaling modules in plants. These allelic differences have important implications for the regulation of gene transcription and protein function in various plant processes, from development to responses to biotic and abiotic stress. Accumulating evidence indicates that naturally diversified calcium signaling is a central molecular adaptation module of natural selection for fine-tuning diverse biological pathways in plants, particularly during signal transduction in response to internal and external changes.

Natural variation in the calcium ion context is responsible for balancing growth/defense trade-offs (Gao et al., 2021, Kolodziej et al., 2021) and tolerance to abiotic stresses (Ye et al., 2018, Zhang et al., 2020) in important models and crop species. The insights gleaned from the natural variation in calcium-mediated signaling offer a complementary understanding of the working mechanisms alongside studies involving artificially induced mutations in laboratory strains. Taken together, these clues could be harnessed to enhance genetic reservoirs for future crop improvement endeavors in light of the changing climate (Alonso-Blanco et al., 2009, Faralli et al., 2019, Liang et al., 2021, Springer and Schmitz, 2017).

## Funding and support

Funding for this study was provided to J.H.K. by the 10.13039/501100003725National Research Foundation of Korea (2021R1A6A1A10042944).

## Author contributions

Y.K. and J.H.K. conceptualized the study and wrote the manuscript. C.D.M.C. contributed to additional synthesis and provided major editorial suggestions.

## Declaration of Competing Interests

The authors declare that they have no known competing financial interests or personal relationships that could have appeared to influence the work reported in this paper.
